# Evaluation of N-terminal Pro-B-Type Natriuretic Peptide (NT-proBNP) as a Prognostic Marker in Patients With Acute Heart Failure in the Emergency Department

**DOI:** 10.7759/cureus.110028

**Published:** 2026-06-01

**Authors:** Tassawwar Iqbal, Abdur Rehman, Ishwa Ehtram Ali, Munazza M Salman, Mohsin Ali Jafri, Usman Nazir, Mateen Ahmed

**Affiliations:** 1 Cardiology, Major Shabbir Sharif Hospital THQ Kunjah, Kunjah, PAK; 2 Cardiology, Punjab Institute of Cardiology, Lahore, PAK; 3 Emergency Department, Hameed Latif Medical Center, Lahore, PAK; 4 Pathology, Avicenna Medical College, Lahore, PAK; 5 General Medicine, Madni Hospital, Gujrat, PAK; 6 Cardiology, Mayo Hospital Lahore, Lahore, PAK

**Keywords:** complications, hospital stay, nt-probnp, patients, predicting

## Abstract

Background: Acute heart failure is a common cause of emergency department admissions and is associated with high morbidity and mortality.

Objective: To evaluate the prognostic value of N-terminal pro-B-type natriuretic peptide (NT-proBNP) in predicting severity and clinical outcomes in patients presenting with acute heart failure in the emergency department.

Methods: This cross-sectional analytical study was conducted at the Department of Cardiology, Punjab Institute of Cardiology, Lahore, Pakistan from January 2025 to July 2025, on 225 patients diagnosed with acute heart failure. NT-proBNP levels were measured at admission. Patients were followed during hospital stay for outcomes including severity of heart failure, need for ICU admission, duration of hospital stay, complications, and in-hospital mortality.

Results: The mean NT-proBNP level was significantly higher in patients with severe heart failure compared to mild to moderate cases (6,920 ± 2,310 pg/mL vs 3,240 ± 1,580 pg/mL, p < 0.001). Patients requiring ICU admission and those with complications or mortality had markedly elevated NT-proBNP levels (p < 0.001). A significant positive correlation was observed between NT-proBNP levels and duration of hospital stay (r = 0.52, p < 0.001). Receiver operating characteristic curve analysis showed good predictive accuracy for severe acute heart failure, with an area under the curve of 0.86 (95% CI: 0.80-0.91; p<0.001). The optimal NT-proBNP cutoff value of 5000 pg/mL was determined using the Youden Index, yielding a sensitivity of 82.1% and specificity of 76.4%.

Conclusion: NT-proBNP is a reliable prognostic marker in acute heart failure, strongly associated with disease severity, adverse outcomes, and prolonged hospitalization. Its use in the emergency department can facilitate early risk stratification and guide clinical decision-making.

## Introduction

Acute heart failure is a typical and life-threatening clinical syndrome that is marked by the onset or acceleration of symptoms and signs of heart failure, which may demand immediate assessment and treatment in the emergency department. It is one of the leading causes of hospitalization in the world and is linked to significant morbidity, mortality, and healthcare usage [1]. Risk stratification in the patient with acute heart failure is crucial in the early stages to inform treatment choices and resource utilization, as well as enhance clinical outcomes. Clinical manifestations of acute heart failure may be unpredictable, with mild dyspnea to severe respiratory discomfort and hemodynamic unstable condition [2]. Standard evaluation techniques such as clinical examination, imaging, and standard laboratory parameters are not necessarily accurate sources of prognostic data, especially at the initial stage of presentation. Thus, more and more reliable and objective biomarkers are needed, which can help clinicians predict the severity of the disease, treatment, and approximate prognosis in the emergency [3].

NT-proBNP is a highly established biomarker secreted by ventricular myocardium in response to the rise in wall stress and volume overload. It is a mirror of the underlying pathophysiological processes of heart failure and has been extensively used as a diagnostic and prognostic tool. NT-proBNP interpretation has diagnostic “grey zones” because levels are influenced by age, BMI, renal function, and other non-cardiac factors. Therefore, this study used receiver operating characteristic (ROC) analysis with the Youden Index to determine the optimal prognostic cut-off [4]. NT-proBNP has been reported to be elevated and correlate with the severity of heart failure, the extent of ventricular dysfunction, and the risk of adverse events, such as hospitalization, and mortality. NT-proBNP has a number of benefits as a prognostic tool in the emergency department. It is fast to determine, is commonly accessible, and offers quantitative data that complements clinical evaluation [5]. A number of studies have also shown that the higher the NT-proBNP levels on presentation, the higher the risk of in-hospital complications, longer hospital stay, intensive care, and short- and long-term death. Furthermore, serial NT-proBNP measurements can offer further understanding of the response to treatment, as well as disease progression [6].

The pathophysiology of acute heart failure is complex and includes interactions of hemodynamic stress, neurohormonal activation, and systemic inflammation [7,8]. Greater ventricular wall stretch causes the release of natriuretic peptides (such as NT-proBNP), which are compensatory responses to stimulate natriuresis, vasodilation, and renin-angiotensin-aldosterone system inhibition. Nevertheless, continuously high levels signify persistent cardiac stress and are related to poor clinical outcomes. This biology justifies the application of NT-proBNP as a diagnostic and prognostic tool in the acute setting [9]. The pressure overload and the expansion of the cardiac ventricle cause the release of a neurohormone called B-type natriuretic peptide (BNP). The property of BNP activation to diagnose left ventricular dysfunction in patients has created significant interest [10]. Although research has shown the levels related to the severity and prognosis of heart failure, BNP was not applicable in active clinical practice until a fast and inexpensive test had been developed [11]. Recent statistics demonstrate that, in this issue of circulation, BNP has now established itself in this area [12].

Objective

The objective was to evaluate the prognostic value of NT-proBNP in predicting severity and clinical outcomes in patients presenting with acute heart failure in the emergency department.

## Materials and methods

This was a cross-sectional analytical study conducted at the Department of Cardiology, Punjab Institute of Cardiology, Lahore, Pakistan from January 2025 to July 2025. A total of 225 patients diagnosed with acute heart failure were included in the study. The sample size was calculated using the WHO sample size calculator. The anticipated sensitivity of NT-proBNP for predicting severe acute heart failure was taken as 82.1%, with a 95% confidence level, 5% margin of error, and expected prevalence of severe acute heart failure based on previous literature. The minimum required sample size was calculated as 225 patients. To ensure inclusion of all eligible patients who presented within the study period, non-probability consecutive sampling was used to recruit all eligible patients who met the inclusion criteria. The study included patients aged 18 years and above who were clinically diagnosed with acute heart failure. Severe renal failure was defined objectively as estimated glomerular filtration rate <30 mL/min/1.73 m² or requirement for dialysis. Sepsis was excluded when patients fulfilled clinical criteria for suspected infection with systemic inflammatory response and had supportive laboratory evidence such as raised procalcitonin or positive cultures, where available. Patients with pulmonary embolism confirmed clinically or radiologically were also excluded.

Data collection

Eligible patients were recruited by receiving approval from the institutional ethical review committee (approval RTPGME-research-047) and informed consent of the participants. Demographic information such as age, gender, and other pertinent comorbidities was obtained. At admission, clinical assessment, such as vital signs and presenting symptoms, was conducted. NT-proBNP levels in the blood were measured by using standard laboratory methods on blood samples collected at presentation. Further tests like electrocardiography, chest radiography and echocardiography were also conducted as a routine clinical care. Patients were followed up during their hospitalization and the outcomes, such as the severity of heart failure, the need to be admitted to an intensive care unit, length of stay, and in-hospital mortality, were recorded. Heart failure severity was classified at presentation using New York Heart Association (NYHA) functional class, clinical status, and echocardiographic findings. Mild heart failure was defined as NYHA class I-II with stable vital signs and no pulmonary edema. Moderate heart failure was defined as NYHA class III with dyspnea on minimal exertion, peripheral edema, or radiographic congestion without shock. Severe heart failure was defined as NYHA class IV or acute pulmonary edema, oxygen saturation <90%, systolic blood pressure <90 mmHg, need for ICU admission/inotropic support, or left ventricular ejection fraction <30%. The main finding was that the NT-proBNP levels were prognostically significant in the severity of acute heart failure. Secondary outcomes were the necessity to be admitted to ICU, length of hospital stay, and in-hospital mortality. Complications assessed during hospital stay included arrhythmias, cardiogenic shock, acute kidney injury or renal failure, respiratory failure requiring ventilatory support, hypotension requiring inotropic support, ICU admission, and in-hospital mortality.

Statistical analysis

Data were analyzed using SPSS version 26.0 (IBM Corp., Armonk, NY, USA). Quantitative variables were expressed as mean ± standard deviation or median with interquartile range, depending on data distribution. Qualitative variables were presented as frequencies and percentages. Normality of continuous variables, including NT-proBNP levels, was assessed using the Shapiro-Wilk test. As NT-proBNP is commonly right-skewed, non-normally distributed values were analyzed using the Mann-Whitney U test or log-transformed before parametric testing, where appropriate. Multivariable logistic regression analysis was performed to assess whether NT-proBNP independently predicted severe acute heart failure after adjustment for age, estimated glomerular filtration rate, hypertension, diabetes mellitus, ischemic heart disease, and body mass index. Categorical variables were compared using chi-square test or Fisher’s exact test where appropriate. To determine whether NT-proBNP independently predicted severe acute heart failure and adverse clinical outcomes, multivariable logistic regression analysis was performed after adjusting for clinically relevant confounders, including age, body mass index, estimated glomerular filtration rate, hypertension, diabetes mellitus, and ischemic heart disease. Adjusted odds ratios with 95% confidence intervals were reported. For time-related outcomes, Cox proportional hazards regression was considered where applicable. A p-value ≤0.05 was considered statistically significant.

## Results

The study included 225 patients with a mean age of 58.4 ± 12.6 years, showing a predominance of male patients (138, 61.3%) compared to females (87, 38.7%). Dyspnea was the most common presenting symptom, reported in 205 (91.1%) patients, followed by peripheral edema in 143 (63.5%) and fatigue in 118 (52.4%). Among comorbid conditions, hypertension was the most prevalent (155, 68.9%), followed by ischemic heart disease (122, 54.2%) and diabetes mellitus (105, 46.7%) (Table 1).

**Table 1:** Baseline Demographic and Clinical Characteristics (n = 225)

Variable	Category	Total (n=225) n (%) / Mean ± SD
Age (years)	-	58.4 ± 12.6
Gender	Male	138 (61.3%)
	Female	87 (38.7%)
Presenting Symptom	Dyspnea	205 (91.1%)
	Peripheral Edema	143 (63.5%)
	Fatigue	118 (52.4%)
Comorbidities	Hypertension	155 (68.9%)
	Diabetes Mellitus	105 (46.7%)
	Ischemic Heart Disease	122 (54.2%)

The mean NT-proBNP level in the severe group was 6,920 ± 2,310 pg/mL, whereas it was 3,240 ± 1,580 pg/mL in the mild to moderate group, demonstrating a highly significant difference (p < 0.001) (Table 2).

**Table 2:** N-terminal Pro-B-Type Natriuretic Peptide (NT-proBNP) Levels According to Disease Severity Statistical comparison performed using independent sample t-test.

Severity Group	n	NT-proBNP (pg/mL) Mean ± SD	t-value	p-value
Mild-Moderate	143	3,240 ± 1,580	11.42	<0.001
Severe	82	6,920 ± 2,310		

Patients requiring ICU admission had markedly elevated NT-proBNP levels (7,150 ± 2,420 pg/mL) compared to those not requiring ICU care (3,780 ± 1,690 pg/mL), with a statistically significant difference (p < 0.001). Similarly, patients who developed complications had higher NT-proBNP levels (7,420 ± 2,510 pg/mL) compared to those without complications (3,610 ± 1,720 pg/mL) (p < 0.001). Mortality was also strongly associated with elevated NT-proBNP, with deceased patients showing levels of 7,880 ± 2,630 pg/mL versus 3,950 ± 1,740 pg/mL in survivors (p < 0.001) (Table 3).

**Table 3:** N-terminal Pro-B-Type Natriuretic Peptide (NT-proBNP) Levels and Clinical Outcomes Statistical comparisons were performed using independent sample t-test.

Outcome	Category	n	NT-proBNP (pg/mL) Mean ± SD	t-value	p-value
ICU Admission	Yes	62	7,150 ± 2,420	10.38	<0.001
	No	163	3,780 ± 1,690		
Complications	Yes	74	7,420 ± 2,510	11.02	<0.001
	No	151	3,610 ± 1,720		
Mortality	Yes	28	7,880 ± 2,630	9.47	<0.001
	No	197	3,950 ± 1,740		

A significant association was observed between NT-proBNP levels and duration of hospital stay. Patients with high NT-proBNP levels (>5000 pg/mL) had a longer mean hospital stay (7.8 ± 2.6 days) compared to those with lower levels (≤5000 pg/mL), who had a mean stay of 4.3 ± 1.8 days (p < 0.001) (Table 4).

**Table 4:** N-terminal Pro-B-Type Natriuretic Peptide (NT-proBNP) and Duration of Hospital Stay Statistical comparison performed using independent sample t-test.

NT-proBNP Group	n	Mean Hospital Stay (days) Mean ± SD	t-value	p-value
High (>5000 pg/mL)	94	7.8 ± 2.6	9.21	<0.001
Low (≤5000 pg/mL)	131	4.3 ± 1.8		

**Figure 1:**
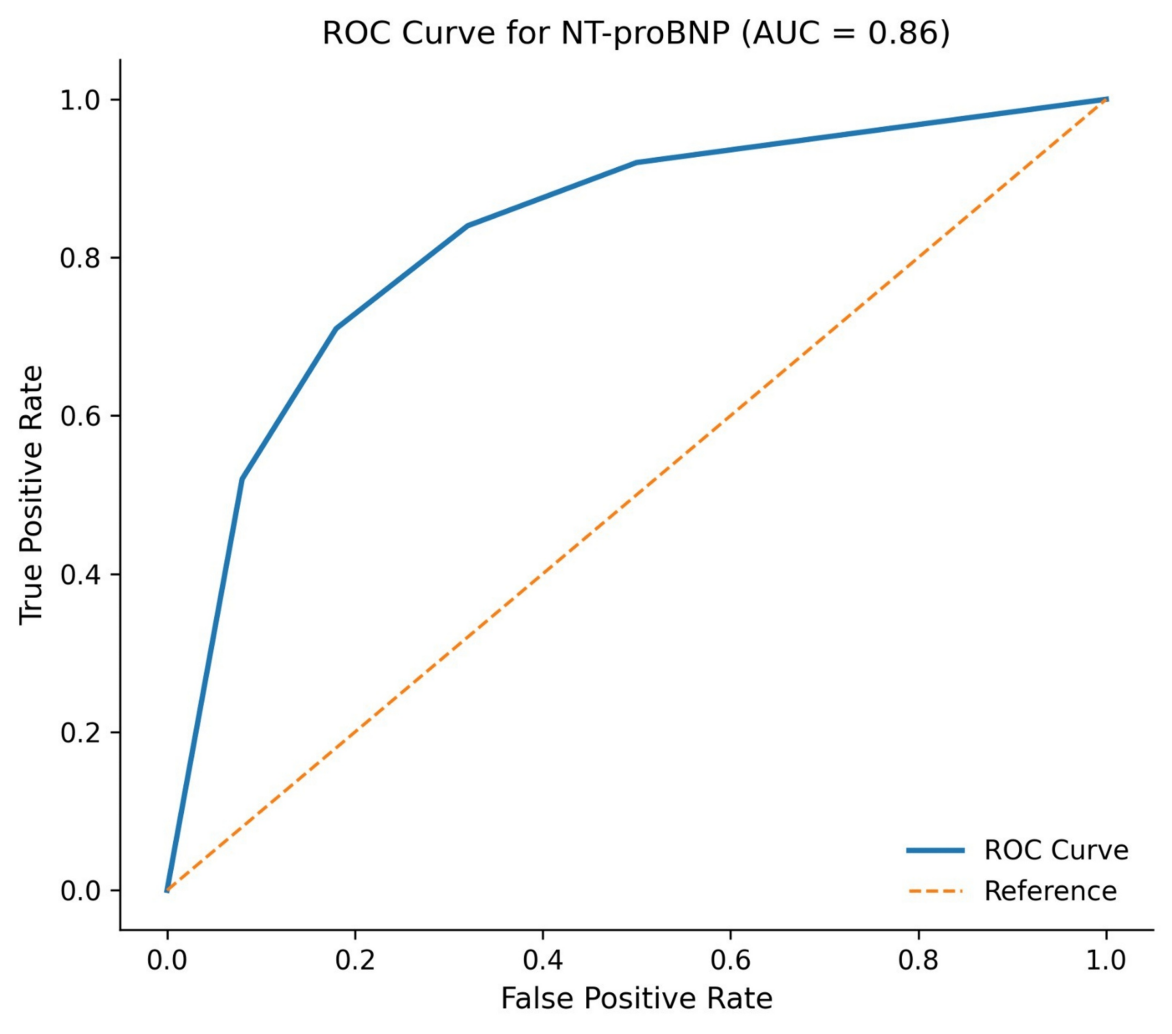
Receiver Operating Characteristic (ROC) Curve Showing Diagnostic Accuracy of N-terminal Pro-B-Type Natriuretic Peptide (NT-proBNP) for Predicting Severe Acute Heart Failure in Emergency Department Patients

Receiver operating characteristic curve analysis demonstrated good predictive accuracy of NT-proBNP for severe acute heart failure, with an area under the curve (AUC) of 0.86 (95% CI: 0.80-0.91). At a cut-off value of 5000 pg/mL, NT-proBNP showed a sensitivity of 82.1% and specificity of 76.4% (Table 5, Figure 1).

**Table 5:** Diagnostic Accuracy of N-terminal Pro-B-Type Natriuretic Peptide (NT-proBNP) The p-value (<0.001) corresponds to statistical significance of the receiver operating characteristic (ROC) analysis (area under the curve (AUC) significantly different from 0.5).

Parameter	Value	p-value
Cut-off Value	5000 pg/mL	-
Sensitivity	0.821	-
Specificity	0.764	-
AUC (95% CI)	0.86 (0.80-0.91)	<0.001

Male patients were more frequent in the severe heart failure group (69.5%) compared with the mild-moderate group (56.6%), although this difference was not statistically significant (χ²=3.74, p=0.053). Hypertension was significantly more prevalent among patients with severe heart failure, present in 78.0% compared with 63.6% in the mild-moderate group (χ²=5.02, p=0.025). Similarly, diabetes mellitus was observed more frequently in the severe group (57.3% vs 40.6%; χ²=5.79, p=0.016), while ischemic heart disease was also significantly associated with severe heart failure, occurring in 64.6% of severe cases compared with 48.3% of mild-moderate cases (χ²=5.45, p=0.020) (Table 6).

**Table 6:** Association of Heart Failure Severity With Categorical Clinical Variables Statistical comparisons were performed using chi-square test.

Variable	Category	Mild–Moderate (n=143) n (%)	Severe (n=82) n (%)	Chi-square	p-value
Gender	Male	81 (56.6)	57 (69.5)	3.74	0.053
	Female	62 (43.4)	25 (30.5)		
Hypertension	Yes	91 (63.6)	64 (78.0)	5.02	0.025
	No	52 (36.4)	18 (22.0)		
Diabetes Mellitus	Yes	58 (40.6)	47 (57.3)	5.79	0.016
	No	85 (59.4)	35 (42.7)		
Ischemic Heart Disease	Yes	69 (48.3)	53 (64.6)	5.45	0.02
	No	74 (51.7)	29 (35.4)		

## Discussion

This focus area assessed the prognostic value of NT-proBNP in patients who present with acute heart failure in the emergency department and found that there was a significant correlation between high levels of NT-proBNP and poor clinical outcomes. The results suggest that NT-proBNP is a valid predictor of early risk stratification with higher levels associated with a higher disease severity, a high probability of ICU stay, a long hospital stay, and a high level of in-hospital mortality. The NT-proBNP level in this group of 225 patients was significantly elevated in patients having severe acute heart failure as opposed to patients with mild to moderate disease. The given observation is in line with the established pathophysiologic role of NT-proBNP, secreted because of the stress and volume overload of the ventricular walls. The significant increase in NT-proBNP of critically ill patients justifies its use as a surrogate endpoint of cardiac dysfunction and hemodynamic decompensation [12,13].

The correlation of higher levels of NT-proBNP with ICU admission in the case of this study points to its usefulness in early triage. The biomarker levels in patients who needed intensive care were much higher at presentation, indicating that NT-proBNP can serve as an early warning of clinical decline. Likewise, patients who acquired complications or in-hospital mortality showed significantly higher levels of NT-proBNP than did patients with positive outcomes [14]. These results underscore the prognostic value of NT-proBNP as a predictor of the short-term outcomes in acute heart failure. NT-proBNP levels were also significantly positively correlated with length of stay, which implies that an increase in biomarker levels is related to more disease burden and slower clinical recovery. The connection also adds to the value of NT-proBNP as a measure not only of the severity of the disease but also of the healthcare resource use [15].

NT-proBNP showed good diagnostic performance in this study with an area under the ROC curve of 0.86, which implies good predictive ability [16]. The cut-off value of 5000 pg/mL was identified to give an optimal sensitivity and specificity to severe disease prediction. The results are consistent with other existing literature, which has also found a comparable diagnostic accuracy of NT-proBNP in acute heart failure patients. Past studies have uniformly demonstrated that NT-proBNP is a powerful predictor of death and unfavorable eventualities, and should be incorporated in clinical decision-making algorithms [17]. Although hypertension, diabetes mellitus, and ischemic heart disease were significantly more common among patients with severe acute heart failure, these comorbidities may themselves influence NT-proBNP levels and adverse outcomes. Therefore, unadjusted comparisons alone cannot establish whether elevated NT-proBNP independently predicts severity or simply reflects a higher-risk clinical phenotype. Adjustment for age, renal function, BMI, and major comorbidities is necessary to determine the independent prognostic value of NT-proBNP.

Limitations

There are a number of limitations to this study. As it was conducted at a single center without external validation, the proposed NT-proBNP cut-off value of 5000 pg/mL should be interpreted as an institution-specific prognostic threshold rather than a universally applicable value. Natriuretic peptide levels may vary according to age, renal function, body mass index, assay calibration, and population characteristics. Multivariable adjustment for important confounders such as age, BMI, estimated glomerular filtration rate, hypertension, diabetes mellitus, and ischemic heart disease was not performed, so the observed associations may reflect a higher-risk clinical phenotype rather than the independent predictive ability of NT-proBNP alone. Only a single admission NT-proBNP value was measured, and serial biomarker monitoring was not performed, limiting assessment of treatment response and discharge readiness. Comprehensive echocardiographic parameters were also not available for all patients. In addition, the study assessed only in-hospital outcomes and did not evaluate 30-day readmission, recurrent emergency visits, or long-term mortality. Due to the observational design and residual confounding, the findings should be interpreted as descriptive associations rather than definitive evidence of independent prognostic performance.

## Conclusions

NT-proBNP levels at admission were significantly associated with acute heart failure severity, ICU admission, complications, prolonged hospital stay, and in-hospital mortality. However, these findings should be interpreted cautiously because NT-proBNP is influenced by age, renal function, body mass index, and other comorbid conditions. The proposed cut-off value of 5000 pg/mL was derived from ROC analysis in this single-center cohort and should be considered an institutional prognostic threshold rather than a universal value. Although the AUC was 0.86 with good discrimination, external validation, likelihood ratios, negative predictive value, serial NT-proBNP measurements, and multivariable adjustment are needed before this threshold can be recommended for routine emergency triage.
